# Acute Hyperthermia Inhibits TGF-β1-induced Cardiac Fibroblast Activation via Suppression of Akt Signaling

**DOI:** 10.1038/s41598-018-24749-6

**Published:** 2018-04-19

**Authors:** Masatoshi Narikawa, Masanari Umemura, Ryo Tanaka, Takayuki Fujita, Utako Yokoyama, Tomoaki Ishigami, Kazuo Kimura, Kouichi Tamura, Yoshihiro Ishikawa

**Affiliations:** 1 Cardiovascular Research Institute, Yokohama City University School of Medicine, Yokohama, Japan; 2 Medical Science and Cardiorenal Medicine, Yokohama City University School of Medicine, Yokohama, Japan

## Abstract

Transforming growth factor-β1 (TGF-β1) induces phenotypic changes in fibroblasts to become myofibroblasts with increased production of extracellular matrix (ECM) components and cytokines. It is also known that excessive activation of myofibroblasts accelerates cardiac fibrosis, remodeling, and thus cardiac dysfunction. However, no effective therapy has been established to prevent this process although recent clinical studies have demonstrated the effectiveness of hyperthermia in cardiac dysfunction. The aim of this study was to examine the molecular mechanism of hyperthermia on TGF-β1-mediated phenotypic changes in cardiac fibroblasts. TGF-β1 increased the expression of IL-6, α-smooth muscle actin (α-SMA), and collagen in human cardiac fibroblasts (HCFs). Hyperthermia (42 °C) significantly prevented these changes, *i.e*., increases in IL-6, α-SMA, and collagen, as induced by TGF-β1 in a time-dependent manner. Immunoblotting showed that hyperthermia decreased Akt/S6K signaling, but did not affect Smad2 and Smad3 signaling. Pharmacological inhibition of Akt signaling mimicked these effects of hyperthermia. Furthermore, hyperthermia treatment prevented cardiac fibrosis in Ang II infusion mice model. Putting together, our findings suggest that hyperthermia directly inhibits TGF-β-mediated activation of HCFs via suppressing Akt/S6K signaling.

## Introduction

Cardiac fibrosis is involved in the pathophysiology of a variety of cardiac diseases, including hypertrophy, heart failure, and arrhythmias, which may result in death^1^. As many as 70% of all myocardial cells are non-myocyte cells, including cardiac fibroblasts, endothelial cells and vascular smooth muscle cells^2^. Among them, fibroblasts are involved in production of extracellular matrix (ECM) proteins^3,4^. Further, in response to various stresses, factors such as transforming growth factor-β1 (TGF-β1), interleukin-6 (IL-6), interleukin-13 (IL-13), platelet-derived growth factor (PDGF) and insulin-like growth factor (IGF) are released and induce phenotypic change of fibroblasts to myofibroblasts, which synthesize larger amounts of ECM components as well as cytokines to heal the injury^5–7^. However, excessive activation of myofibroblasts induces ECM accumulation and cardiac remodeling, leading to cardiac dysfunction.

TGF-β1 regulates cell proliferation, apoptosis, migration, and synthesis of ECM components, such fibronectin and collagen, in the heart^8^. It plays a pivotal role in cardiac hypertrophy and cardiac fibrosis by activating fibroblasts and inducing collagen production^9–11^.

IL-6 is a major inflammatory cytokine that was originally identified as B-cell-stimulating factor 2 (BSF-2) in culture supernatant of mitogen- or antigen-stimulated peripheral blood mononuclear cells^12^. IL-6 level is highly correlated with severity of heart failure, myocardial infarction and cardiac fibrosis^13,14^. Cardiac fibroblasts are the major source of IL-6, compared with cardiomyocytes and endothelial cells, in heart tissue^15^. IL-6 induces the development of inflammation and cardiac remodeling^16,17^. Moreover, Seong *et al*. reported that TGF-β induced α-smooth muscle actin (α-SMA) expression in Tenon’s fibroblasts via IL-6 production^18^. Zhou *et al*. showed that IL-6 regulated α-SMA expression in cardiac fibroblasts stimulated with angiotensin (Ang)?^19^.

Waon therapy (an infrared warming therapy) is a non-pharmacological therapy that has improved the outcome of patients with chronic heart failure^20^. It is suggested to improve vascular endothelial function, hemodynamics and sympathetic nervous system function^21^. Wakisaka *et al*. also demonstrated that hyperthermia treatment is effective in suppressing Ang II-mediated atrial fibrosis via induction of heat shock protein (HSP)−72^22^.

However, the function and cellular signaling pathway of hyperthermia in cardiac fibroblasts remain elusive. In the present work, we examined the effects of hyperthermia on cardiac fibroblasts and we also investigated the signaling mechanism. We show that hyperthermia suppresses TGF-β1-induced IL-6 production, α-SMA expression and collagen synthesis in human cardiac fibroblasts (HCFs), resulting in inhibition of both inflammatory response and differentiation of HCFs into myofibroblasts, most probably via Akt/S6K signaling. Our findings support the idea that hyperthermia could be an effective non-pharmacological treatment option for preventing cardiac fibrosis.

## Materials and Methods

### Reagents

Reagents were purchased from Sigma-Aldrich (MO, USA) unless otherwise specified. Antibodies used in western blotting and immunofluorescence staining are described in each section. Human TGF-β1 was purchased from Sigma-Aldrich (MO, USA). LY294002 was purchased from Cell Signaling (MA, USA). KNK 437 was purchased from Abcam (Tokyo, JAPAN).

### Cell culture and hyperthermia treatment

Human cardiac fibroblasts (HCFs) were purchased from ScienCell Research Laboratories (CA, USA) and maintained in FM-2 medium supplemented with 1% penicillin-streptomycin, 1% FGS-2 and 2% fetal bovine serum (FBS)^23^. All cells were maintained in a humidified 5% CO_2_ atmosphere at 37 °C. In general, HCFs from passages 4–8 were used. Prior to treatment, culture medium was replaced with serum-deprived (0.5% FBS) medium for 3 hours^24^. Cells were treated with TGF-β1 (0.2 to 5 ng/ml). Untreated cells cultured under similar conditions served as controls. Following 24 h of treatment, culture medium was collected and IL-6 was determined by ELISA^25^. Cells were immediately subjected to protein and RNA extraction. In the hyperthermia (HT) treatment study, cells ware maintained in a humidified 5% CO_2_ atmosphere at 42 °C (hyperthermia) or 39 °C (mild hyperthermia).

### Membrane microarray

Effect of hyperthermia on mRNA expression profiling was evaluated by microarray analysis. HCFs ware maintained in 37 (control) or 42 °C (Hyperthermia) for 30 min or 60 min followed by total RNA was extracted from HCFs 6 h after hyperthermia treatment. Microarray experiments were carried out using SurePrint G3 Human GE 8 × 60 K v3 Microarray (Agilent technologies, CA, USA) with total RNA as starting material according to the manufacturer’s protocol.

### Cell viability assay

Cell proliferation assay was done with a commercial kit, 2,3-bis(2-methoxy-4-nitro-5-sulfophenyl)−5-[(phenylamino)carbonyl]−2H-tetrazolium inner salt (XTT) Cell Proliferation Assay Kit (ATCC, VA, USA)^26^.

### Immunofluorescence staining

Immunofluorescence staining was performed as previously described^27^. Briefly, cells were grown to 80% confluence on 12 mm coverslips, washed with PBS, and fixed with 4% formaldehyde for 15 min. The coverslips were pre-incubated for 30 min at room temperature in 0.01% Triton X-100 (PBS-Triton), then the cells were treated with primary antibody (anti-α-SMA (Sigma, 1:400)) overnight at 4 °C and thereafter with Alexa Fluor 488 goat anti-mouse IgG (Invitrogen,1:1000) as the secondary antibody for 1 h. After extensive washing in PBS, cells were stained with 4′,6-diamidino-2-phenylindole (DAPI) (Invitrogen, 1:5000) to detect cell nuclei. Cells were then visualized by fluorescence microscopy using an inverted microscope (Nikon). Images were obtained by digital capture and quantified with Image J software (NIH).

### Western blotting

Western blot analyses were performed as previously described^26,28^. Briefly, cells were lysed and sonicated in RIPA buffer (Thermo Scientific, IL, USA). Equal amounts of protein were subjected to sodium dodecyl sulfate polyacrylamide gel electrophoresis (SDS-PAGE). After electrophoretic separation, protein bands were transferred to Millipore Immobilon-P membrane followed by immunoblotting with antibodies against molecules of interest. The following primary antibodies were used for immunoblotting: anti-Smad2 (Cell Signaling, MA, USA, 1:1000), anti-p-Smad2 (Cell Signaling, 1:1000), anti-Smad3 (Cell Signaling, 1:1000), anti-p-Smad3 (Cell Signaling, 1:1000), anti-Akt (Cell Signaling, 1:1000), anti-p-Akt (Thr308 and Ser473) (Cell Signaling, 1:1000), anti-S6K (Cell Signaling, 1:1000), anti-p-S6K (Cell Signaling, 1:1000), anti-α-SMA (SIGMA, 1:2000), anti-GAPDH (Santa cruz Biotechnology, CA, USA, 1:5000) and anti-HSP70 (Invitrogen, 1:1000) antibodies. Chemiluminescence detection was performed using the ECL reagent (Bio-Rad Laboratories. Inc., CA, USA). Signal intensities of bands were quantified with Image J software (NIH).

### Quantitative real-time reverse transcriptase-polymerase chain reaction (RT-PCR)

Isolation of total RNA, generation of cDNA and RT-PCR analysis were done as previously described^28^. The sequences of the specific primers were as follows: IL-6 (forward, 5′-CCAGGAGCCCAGCTATGAA-3′; reverse, 5′-TTCTGCCAGTGCCTCTTTG-3′), ACTA2 (forward, 5′-ATTGCCGACCGAATGCAGA-3′; reverse, 5′-ATGGAGCCACCGATCCAGAC-3′), col1A1 (forward, 5′-CCCGGGTTTCAGAGACAACTTC-3′; reverse, 5′-TCCACATGCTTTATTCCAGCAATC-3′), mouse α-SMA (forward, 5′-GGACGTACAACTGGTATTGTGC-3′; reverse, 5′-TCGGCAGTAGTCACGAAGGA-3′), fibronectin (forward, 5′-GAGAATAAGCTGTACCATCGCAA-3′; reverse, 5′-CGACCACATAGGAAGTCCCAG-3′), MMP-2 (forward, 5′-GATACCCCTTTGACGGTAAGGA-3′; reverse, 5′-CCTTCTCCCAAGGTCCATAGC-3′) and GAPDH (forward, 5′-CCCATCACCATCTTCCAGGAGCG-3′; reverse, 5′-GGCAGGGATGATGTTCTGGAGAGCC-3′)^25^. PCR consisted of an initial cycle of 95 °C for 30 sec, then 40 cycles, each consisting of denaturation at 95 °C for 5 sec, followed by annealing and primer extension at 60 °C for 30 sec. Melting curve analysis was done from 60 °C to 95 °C with a heating rate of 0.3 °C per sec. The abundance of each gene was determined relative to that of GAPDH transcript.

### Measurement of IL-6 protein by enzyme-linked immuno-sorbent assay (ELISA)

The production of interleukin-6 (IL-6) in the presence of TGF-β1 for 24 h was measured in HCFs using a human IL-6 quantitative ELISA kit (R&D Systems Inc., MN, USA) according to the manufacturer’s instructions^25^.

### Angiotensin II infusion model and hyperthermia treatment

Eight-week-old male C57BL/6 mice were purchased from Japan SLC (Shizuoka, Japan). These mice were randomly assigned into three groups as follows: normal saline (NS), Ang II infusion (AT) or Ang II infusion with hyperthermia treatment (AT + heat). Under isoflurane anesthesia (2%), a mini-osmotic pump (ALZET, model 2002; DURECT Corporation, CA, USA) filled with either Ang II (1.5 μg/kg/min; Sigma-Aldrich, MO, USA) or normal saline was inserted underneath the skin^29^. Hyperthermia treatments were performed using a heating plate (NEO HOTPLATE HI-1000; ASONE, Japan) at 42 °C for 60 min per every day and core temperatures were monitored by measurement of the rectal temperature. Body weight (BW), systolic blood pressure (SBP), and heart rate (HR) were measured before, 1 week, and 2 weeks after Ang II infusion. SBP was measured with a non-invasive computerized tail-cuff system (BP-98AL; Softron, Japan). After 2 weeks of Ang II infusion, all mice were euthanized with isoflurane and the heart was rapidly excised. Half of the left ventricle (LV) was fixed in 10% formaldehyde solution for histological staining and total RNA was collected from another half of the LV.

### Histological analysis

LV tissues were fixed with formalin, embedded in paraffin, and sectioned at 3.5-μm thickness. Collagen deposition was evaluated by Sirius Red staining using the Picro-sirius Red Stain Kit (ScyTek Laboratories, UT, USA). Fibrotic area in 4 random fields was measured by Image J Software^30^. Localization of α-SMA was evaluated by immunohistochemical staining with antibody. A color extraction method using Keyence software was performed to quantify expression of α-SMA^25^. Detail of immunohistochemical staining was previously described^28^.

### Ethics statement

Animal experiments were performed according to the Yokohama City University guidelines for experimental animals. The Animal Care and Use Committee at Yokohama City University, School of Medicine, approved all animal studies. All experimental protocols were approved by the Animal Care and Use Committee at Yokohama City University, School of Medicine.

### Data analysis and statistics

Values represent mean ± SEM. Statistical comparisons among groups were performed using Student’s *t*-test, one-factor analysis of variance (ANOVA) or two-way ANOVA with the Bonferroni post hoc test. The criterion of statistical significance was set as *p* < 0.05. Significant differences are indicated by *p < 0.05, **p < 0.01, and ***p < 0.001; ns, not significant.

## Results

### Hyperthermia reduced IL-6 mRNA expression in human cardiac fibroblasts

**Figure 1:**
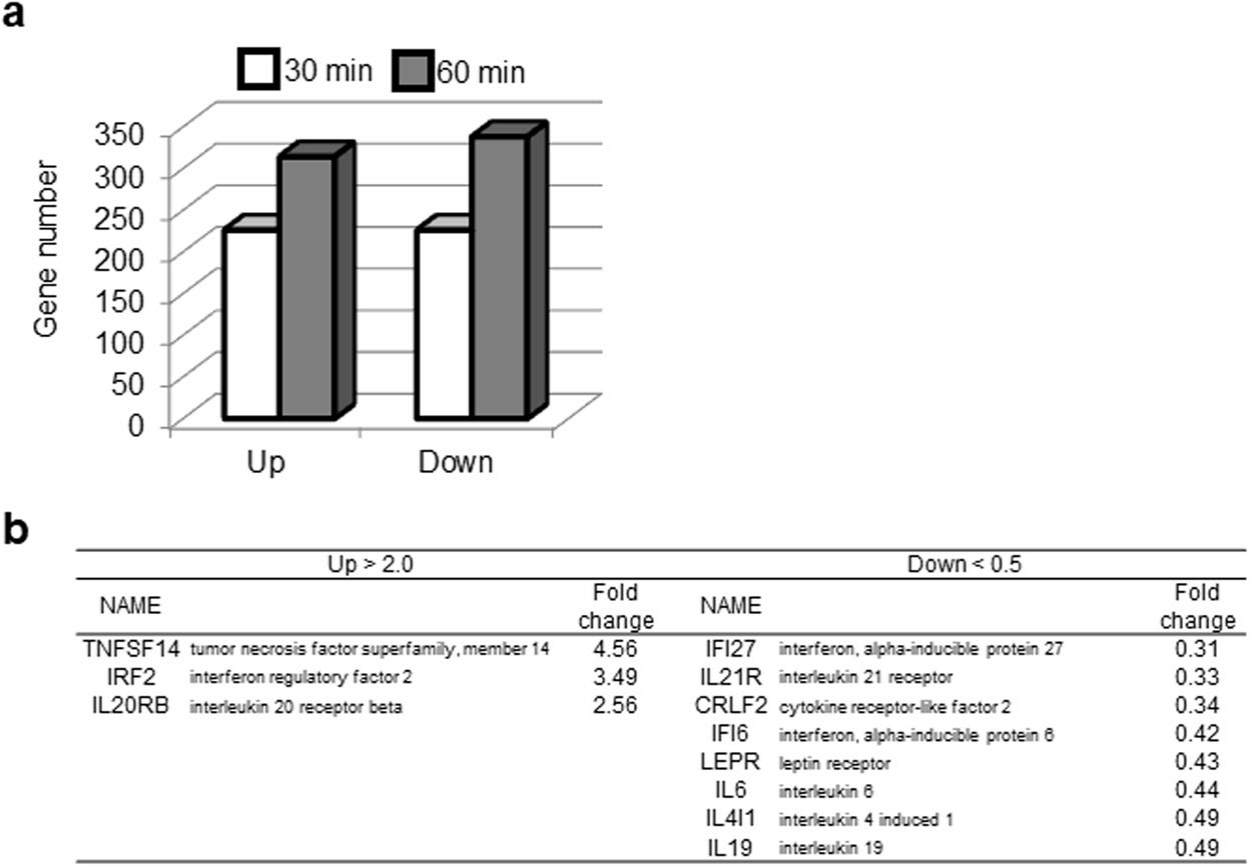
Gene expression changes induced by hyperthermia in human cardiac fibroblasts. (**a**) Histograms show the number of genes whose expression was upregulated (>2.0 fold) or downregulated (<0.5 fold) by hyperthermia treatment at 30 or 60 min. Histogram bars, 30 min (white) and 60 min (gray). (**b**) Cytokine-related genes were extracted from among the genes whose expression was altered.

We performed microarray analysis to investigate the effect of hyperthermia on gene expression in HCFs. Comparison of gene expression levels with/without heat exposure (42 °C) for 30 min, *i.e*. hyperthermia, showed that hyperthermia increased expression of 227 genes and decreased that of 226 genes at 30 min. Furthermore, it increased expression of 314 genes and decreased that of 339 genes at 60 min (Fig. 1a). Among the genes that showed large expression changes (increased 2.0-fold or more, or decreased to 0.5 fold or less), cytokine-associated genes were extracted, and are listed in Fig. 1b. Among these genes, we focused on downregulation of IL-6, because IL-6 is a pleotropic cytokine with a wide range of biological activities related to immune response, hematopoiesis, and inflammation^12^. Elevated IL-6 level is associated with heart failure and is a strong predictor of 1-year mortality^31,32^. It should be noted that hyperthermia significantly reduced cell viability both in the absence and presence of TGF-β1 (Supplemental Fig. 1).

### TGF-β1 increased IL-6 mRNA expression, production and α-SMA protein expression in HCFs

**Figure 2:**
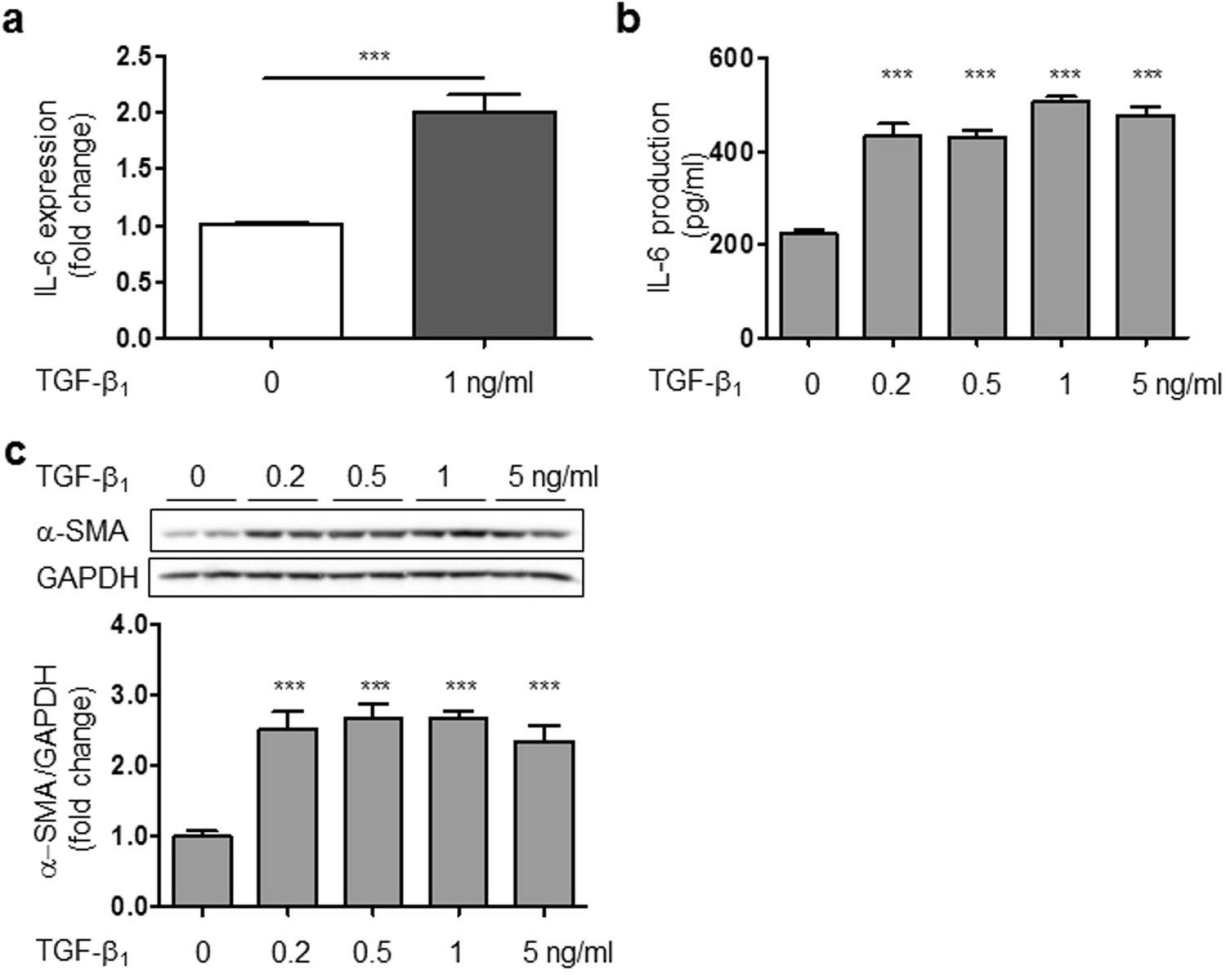
TGF-β1 increased IL-6 mRNA expression, IL-6 production, and α-SMA protein expression in HCFs. (**a**) IL-6 mRNA expression in the presence of TGF-β1 for 6 hours in HCFs (n = 4, ***p < 0.001). (**b**) IL-6 production in the presence of TGF-β1 for 24 h (n = 4, ***p < 0.001). (**c**) TGF-β1-induced increase of α-SMA protein expression after 24 h. (n = 4, ***p < 0.001). The original immunoblot image is shown in Supplemental Fig. 6.

TGF-β plays an important role in the pathogenesis of cardiac remodeling and fibrosis^10^. However, it has not been established whether TGF-β is involved in IL-6 production in HCFs. Therefore, we next examined the effect of TGF-β1 on IL-6 expression in cultured HCFs. TGF-β1 markedly increased IL-6 at the mRNA and protein levels in a dose-dependent manner in HCFs (Fig. 2a,b). It also increased protein expression of α-SMA, which is a molecular marker of differentiated myofibroblasts (Fig. 2c)^33^.

### Hyperthermia (42 °C) inhibited the TGF-β1-induced increase of IL-6 mRNA and production in HCFs

**Figure 3:**
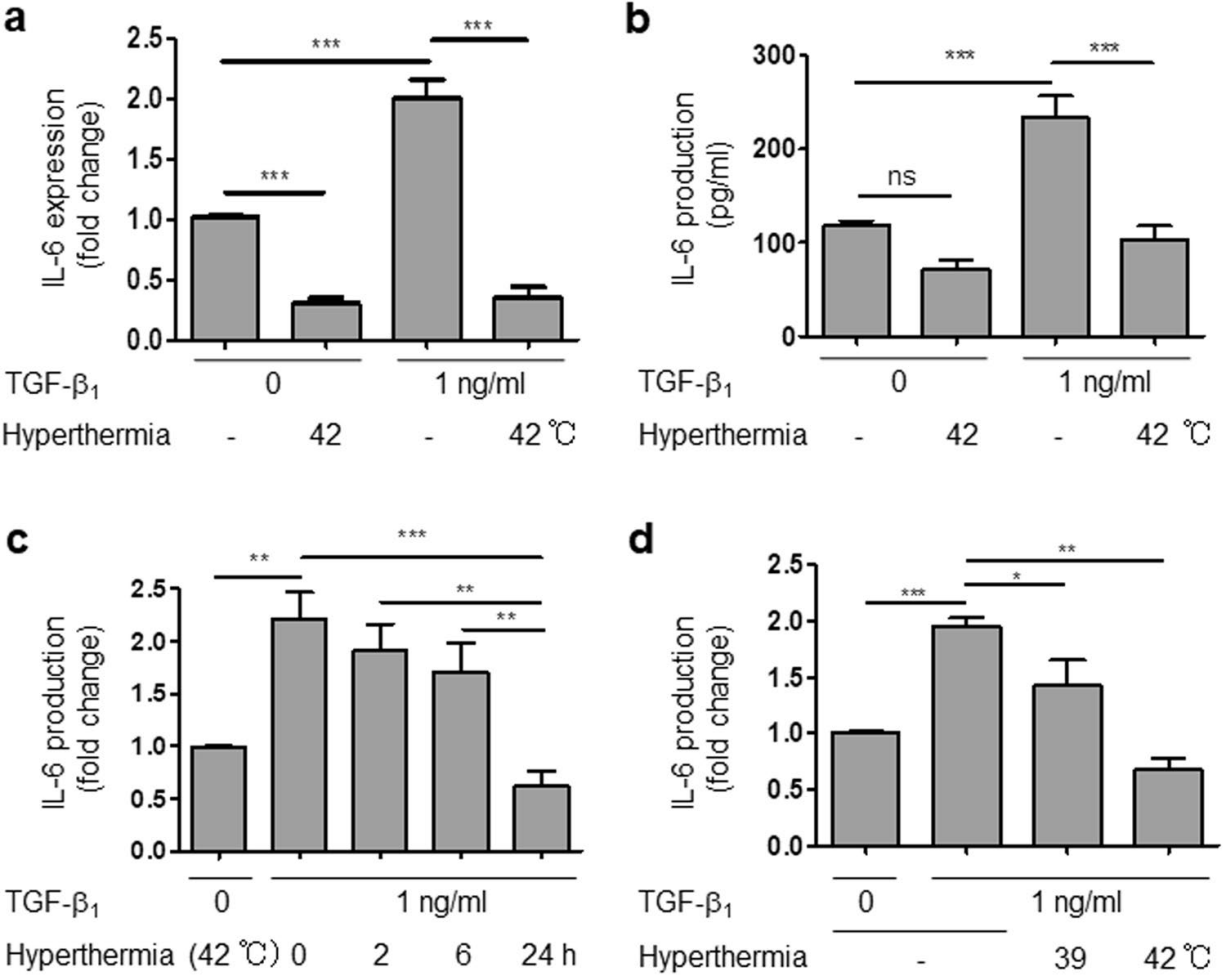
Hyperthermia inhibited IL-6 mRNA and production in HCFs. (**a**) IL-6 mRNA expression in HCFs stimulated by hyperthermia (42 °C) and/or TGF-β1 for 6 h (n = 4, ***p < 0.001). (**b**) IL-6 production from HCFs stimulated by hyperthermia (42 °C) and/or TGF-β1 for 24 h (n = 4, ***p < 0.001, ns: no significant difference). (**c**) Time dependence of the effect of hyperthermia (42 °C) effect on IL-6production in the presence of TGF-β1 for 24 h (n = 4–5, **p < 0.01, ***p < 0.001). (**d**) Effects of hyperthermia (42 °C) and mild hyperthermia (39 °C) on IL-6 production in the presence of TGF-β1 for 24 h (n = 4–8, *p < 0.05, **p < 0.01, ***p < 0.001).

Next, we examined whether hyperthermia influences the TGF-β1-induced increase of IL-6. We found that hyperthermia strongly suppressed the TGF-β1-induced increase of IL-6 at both the mRNA and protein levels (Fig. 3a,b). This inhibitory effect was both time- and temperature-dependent (Fig. 3c,d). These results are consistent with those of microarray analysis, and suggest that hyperthermia may be a powerful therapeutic modality for inhibiting the development of inflammation and cardiac remodeling.

### Hyperthermia inhibited mRNA and protein expression of α-SMA in HCFs

**Figure 4:**
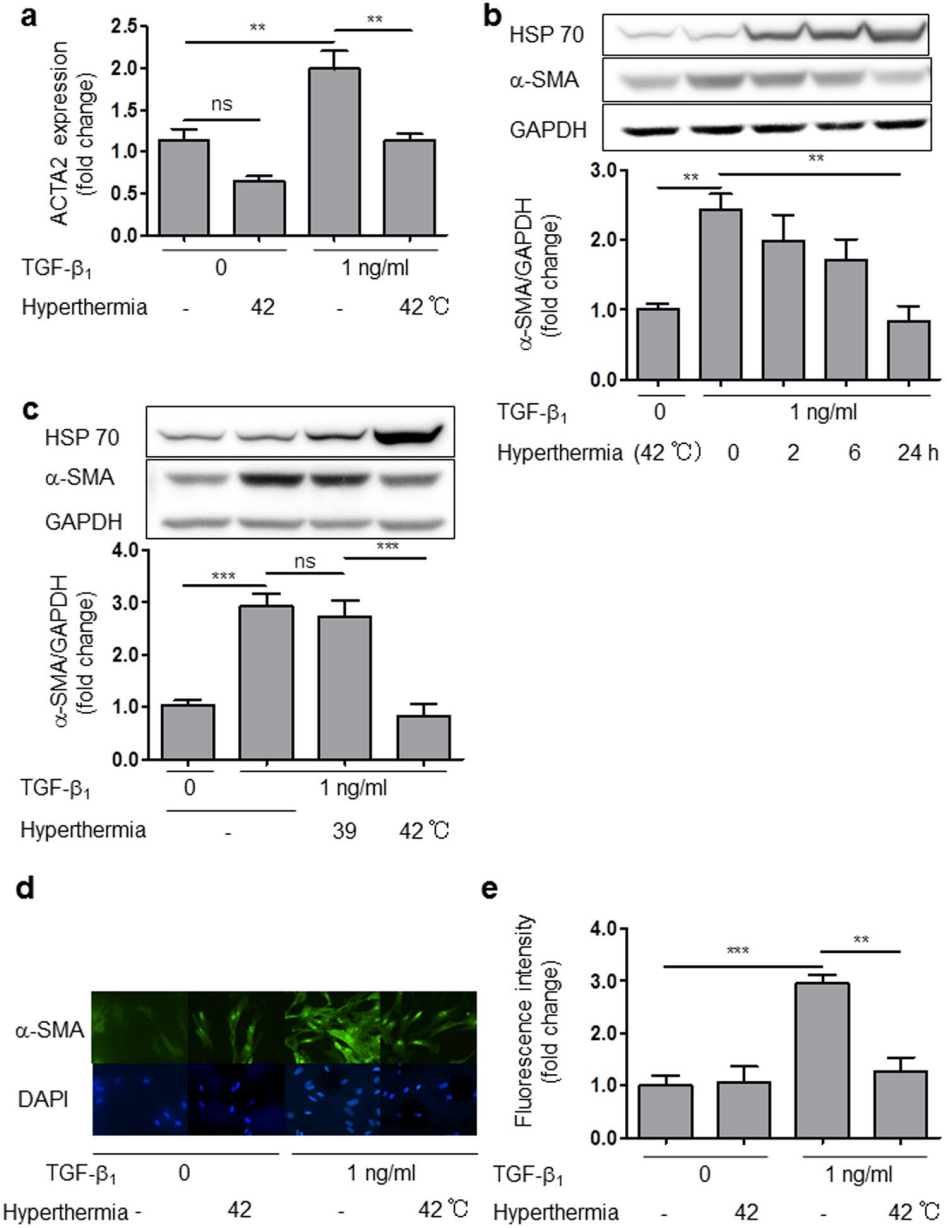
Hyperthermia inhibited α-SMA protein expression in HCFs. (**a**) Hyperthermia (42 °C) inhibited ACTA2 mRNA expression in HCFs with or without TGF-β1 for 24 h (n = 4, **p < 0.01, ns: no significant difference). (**b**) Time dependence of the effect of hyperthermia (42 °C) on TGF-β1-induced α-SMA protein expression over 0–24 h (n = 4–5, **p < 0.01). (**c**) Effect of mild hyperthermia (39 °C) or hyperthermia (42 °C) on TGF-β1-induced α-SMA protein expression at 24 h (n = 4–6, ***p < 0.001, ns: no significant difference). (**d**) Immunocytochemistry of α-SMA in HCFs in the presence of TGF-β1 with or without hyperthermia. (**e**) Fluorescence staining of α-SMA in HCFs in the presence of TGF-β1 with or without hyperthermia (42 °C) (n = 4, **p < 0.01, ***p < 0.001). The original immunoblot images are shown in Supplemental Fig. 7.

Differentiation from fibroblast to the myofibroblast phenotype, which is characterized by expression of smooth muscle actin, is induced by TGF-β1, cytokines, ECM components, and various growth factors^2^. Myofibroblast formation is associated with fibrotic scars in various injury models^34^. TGF-β1 promotes IL-6 expression and induces trans-differentiation of human Tenon’s fibroblasts to myoblasts^35^. Therefore, we hypothesized that TGF-β1 also promotes IL-6 production in HCFs, inducing differentiation from cardiac fibroblasts to myofibroblasts. To test this idea, we examined the effect of TGF-β1 on α-SMA expression and also investigated whether hyperthermia suppressed TGF-β1-induced α-SMA expression in HCFs. Hyperthermia (42 °C) markedly decreased mRNA expression of alpha-actin-2 (ACTA2), which encodes α-SMA (Fig. 4a), and the suppression was time-dependent (Fig. 4b). However, mild hyperthermia (39 °C) had no effect on α-SMA protein expression (Fig. 4c). Hyperthermia (42 °C) had no effect on mRNA of MMP-2 and Fibronectin (Supplemental Fig. 2).

Immunofluorescence images showed that TGF-β1 increased the expression of α-SMA stress fibers and cell size. Interestingly, hyperthermia (42 °C) negated these changes (Fig. 4d,e).

### Hyperthermia attenuated mRNA expression of collagen I

**Figure 5:**
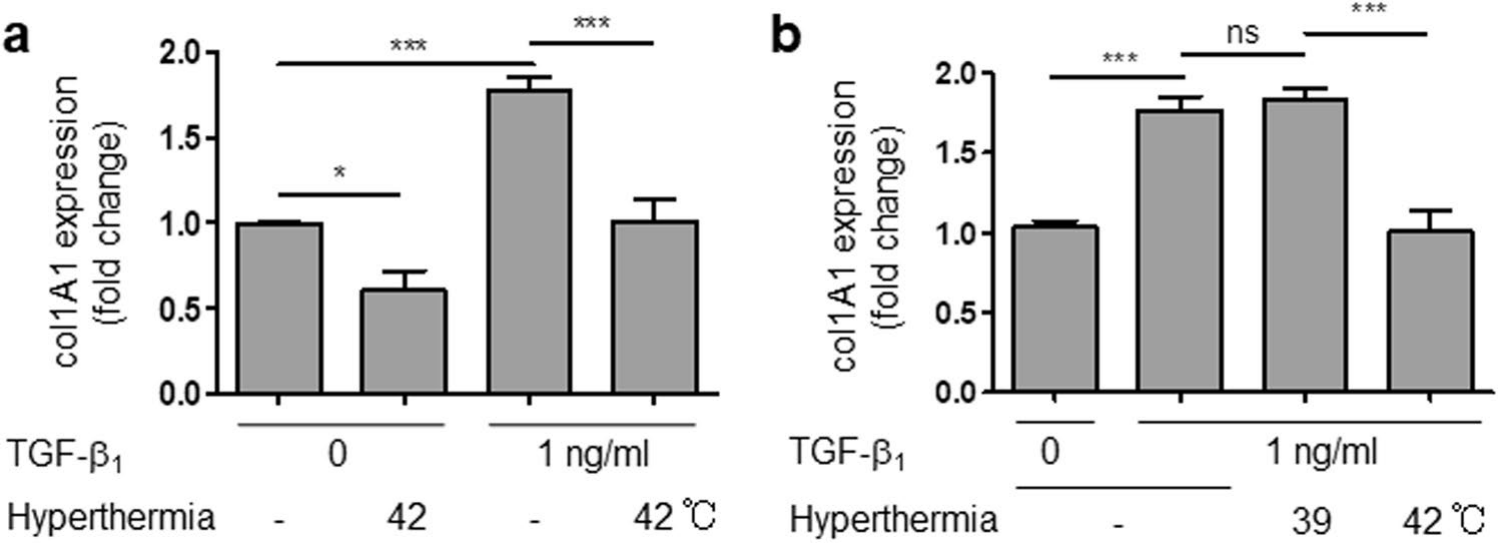
Hyperthermia inhibited collagen synthesis in HCFs. (**a**) Hyperthermia (42 °C) inhibited collagen mRNA expression in HCFs with or without TGF-β1 for 24 h (n = 4–6, *p < 0.05, ***p < 0.001). (**b**) Effects of mild hyperthermia (39 °C) or hyperthermia (42 °C) on TGF-β1-induced collagen mRNA expression for 24 h (n = 4–6, ***p < 0.001, ns: no significant difference).

Hyperthermia (42 °C) also attenuated expression of col1A1 gene, which encodes collagen I protein, in the presence or absence of TGF-β1 (Fig. 5a). In contrast, mild hyperthermia (39 °C) did not affect α-SMA or collagen mRNA levels (Figs 4c and 5b, respectively). Taken together, these findings suggested that hyperthermia at an appropriate temperature (42 °C) has a potent blocking effect on HCFs trans-differentiation and collagen synthesis.

### Hyperthermia suppressed Akt/S6K signaling

**Figure 6:**
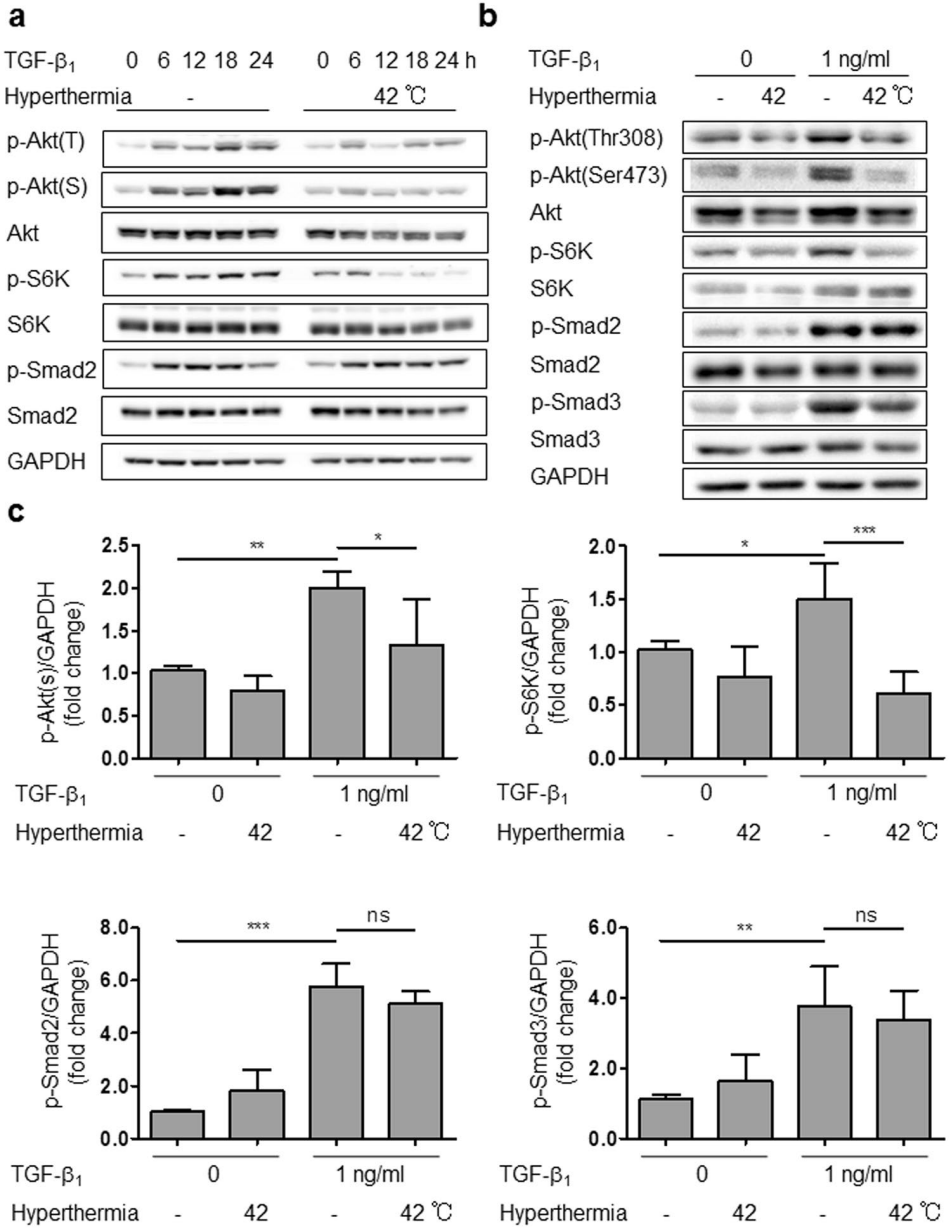
Hyperthermia suppressed Akt/S6K signaling. (**a**) Time course of protein phosphorylation in HCFs stimulated by TGF-β1 with or without hyperthermia for 0 to 24 h. (**b**) Representative western blot of protein phosphorylation in HCFs stimulated by TGF-β1 with or without hyperthermia (42 °C) for 6 h. (**c**) Normalized protein phosphorylation at 6 h determined by densitometry (n = 4–5, *p < 0.05, **p < 0.01, ***p < 0.001, ns: no significant difference). The original immunoblot images are shown in Supplemental Fig. 8.

We next examined the cellular signaling pathway through which hyperthermia attenuates TGF-β1 stimulation in HCFs. TGF/Smad signaling is a canonical pathway leading to fibrotic change^36^. TGF-β1 phosphorylates the serine/threonine kinase Akt (also known as protein kinase B; PKB)^37^. We found that hyperthermia attenuated TGF-β1-induced phosphorylation of Akt (at both Thr308 and Ser473) and ribosomal P-protein S6 kinase (S6K) in a time-dependent manner (Fig. 6a-c). S6K protein is an mTOR pathway effector, and mTOR/S6K signaling stimulates protein synthesis^38^. Interestingly, hyperthermia caused dephosphorylation of Akt after 6 hours, and dephosphorylation of S6K after 12 hours (Fig. 6a-c), indicating that Akt signaling may be located upstream of S6K. In contrast, hyperthermia did not prevent Smad2 and Smad3 phosphorylation.

### Akt inhibitor mimicked the effect of hyperthermia

**Figure 7:**
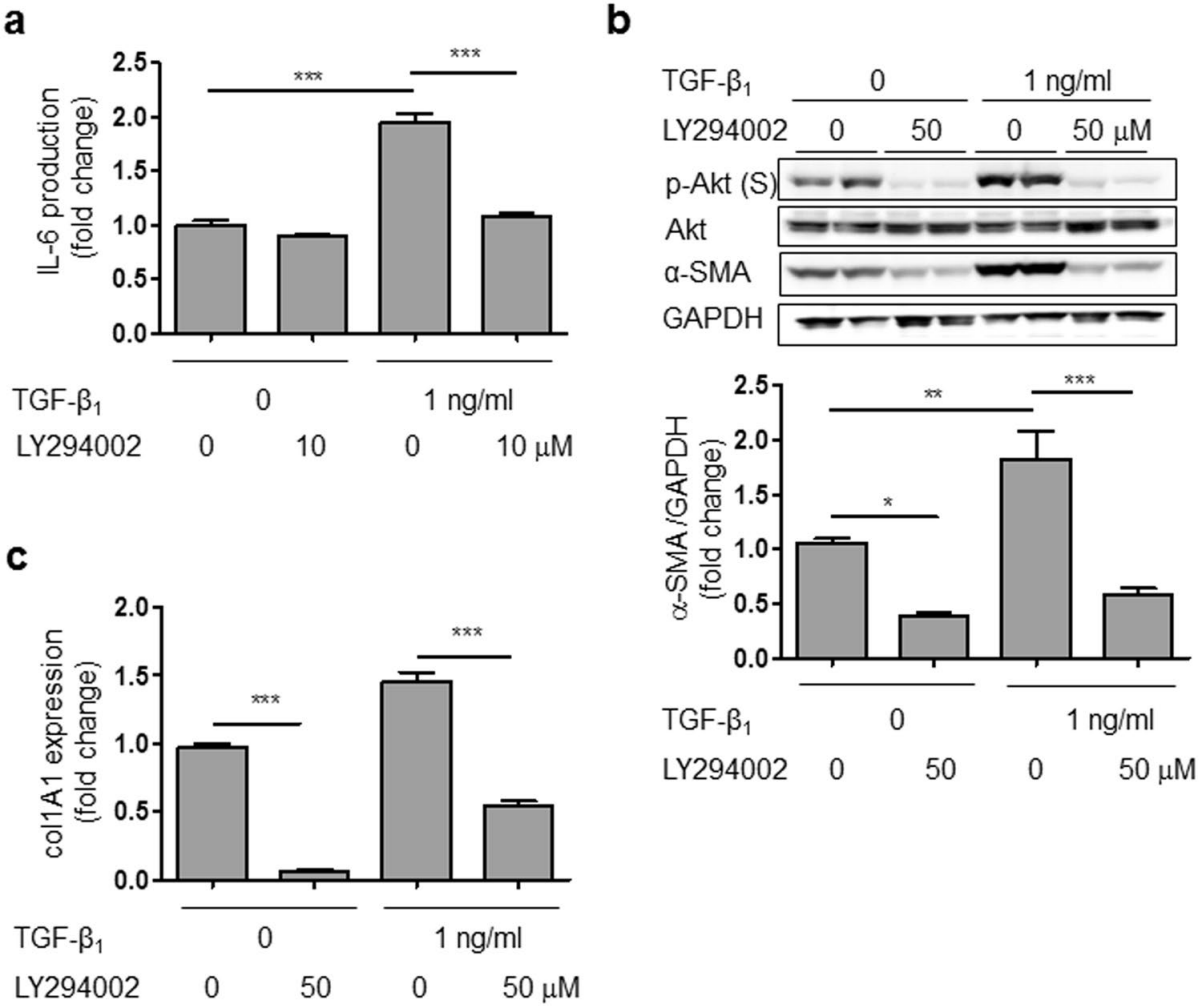
Akt inhibitor attenuated TGF-β1-induced IL-6 production and α-SMA protein expression, and reduced collagen mRNA expression in either the absence or presence of TGF-β1. (**a**) Akt inhibitor (LY294002) decreased TGF-β1-induced IL-6 production in HCFs (n = 4–8, ***p < 0.001). (**b**) Akt inhibitor (LY294002) decreased TGF-β1-induced α-SMA protein expression in HCFs (n = 4, *p < 0.05, **p < 0.01, ***p < 0.001). (**c**) Akt inhibitor (LY294002) decreased collagen mRNA in HCFs in either the absence or presence of TGF-β1 (n = 4, ***p < 0.001). The original immunoblot images are shown in Supplemental Fig. 9.

To investigate the role of Akt signaling in stimulation of HCFs by TGF-β1, we examined whether Akt inhibitor LY294002 has similar effects to hyperthermia on expression of IL-6 and α-SMA. Indeed, LY294002 decreased TGF-β1-induced IL-6 production and α-SMA protein expression in HCFs (Fig. 7a,b). Moreover, Akt inhibitor greatly decreased col1A1 mRNA expression (Fig. 7c). In contrast, HSP inhibitor, KNK437 did not negate the hyperthermia-induced suppression of α-SMA and IL-6 (Supplemental Fig. 3). These results suggested that hyperthermia attenuates fibrotic responses via the Akt/S6K signaling pathway and is independent of HSP70 protein expression, as illustrated in Supplemental Fig. 4.

### Hyperthermia treatment prevented cardiac fibrosis in Ang II infusion mice model

**Figure 8:**
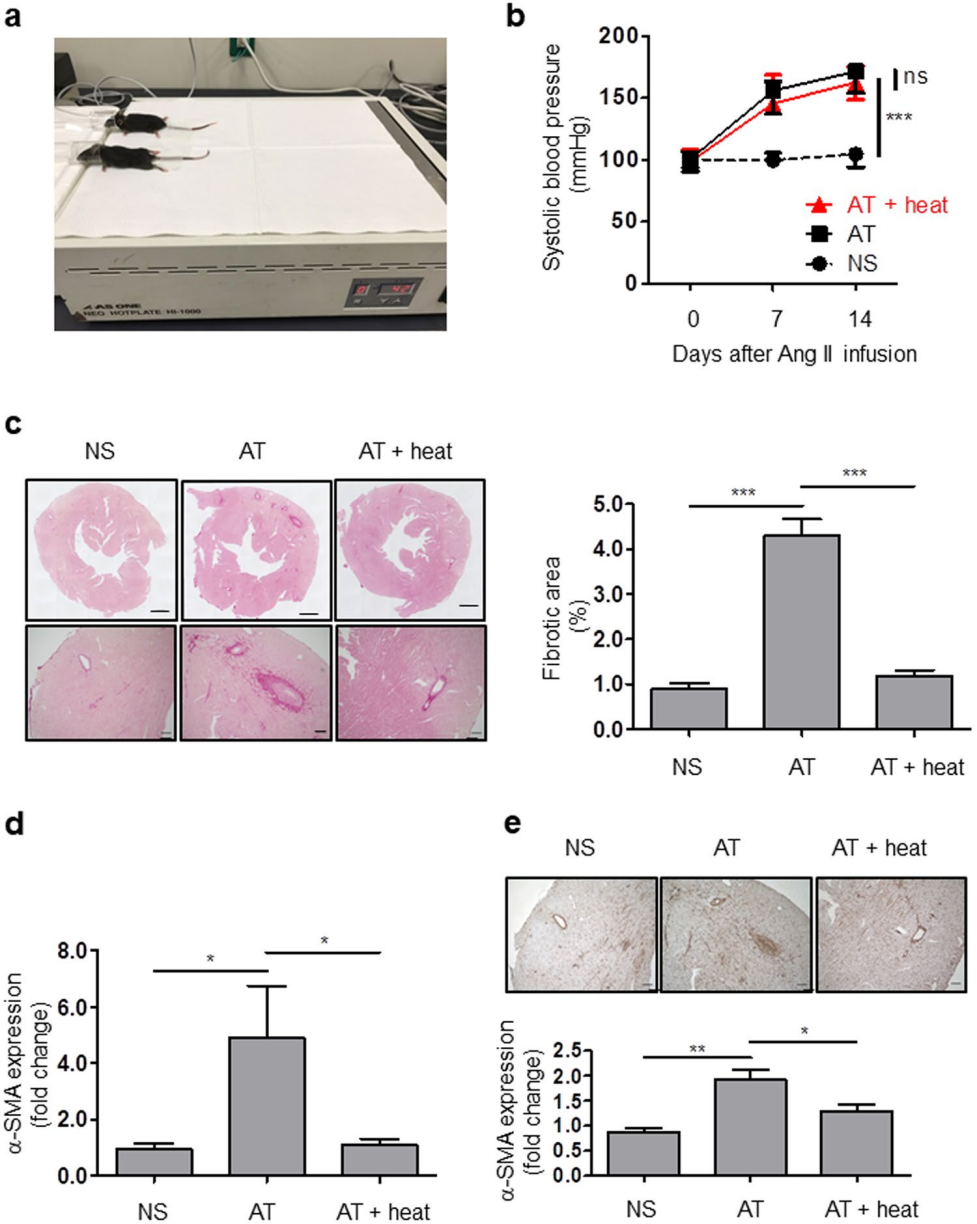
Hyperthermia treatment prevented fibrotic change in angiotensin infusion model. (**a**) Picture of mice undergoing hyperthermia treatment by heating plate. (**b**) Ang II increased systolic blood pressure (SBP) in mice with or without hyperthermia (normal saline (NS), Ang II infusion (AT) and Ang II infusion with hyperthermia treatment (AT + heat)), (n = 5, ***p < 0.001, ns: no significant difference). (**c**) Sirius Red staining of heart sections. Scale bars: 600 μm (*top*), 100 μm (*bottom*). Representative images of perivascular fibrosis (*left*) and quantitative analysis of fibrotic area in left ventricle (LV) (*right*, n = 4–5, ***p < 0.001). (**d**) Hyperthermia attenuated α-SMA mRNA expression in mice LV (n = 5, *p < 0.05). (**e**) Immunohistological staining of heart sections for α-SMA (brown). Scale bars: 100 μm. (n = 4, *p < 0.05, **p < 0.01).

To investigate the effect of hyperthermia treatment on cardiac fibrosis using an *in vivo* model, we evaluated the cardiac fibrosis in the Ang II infusion mice model. Mice core temperatures were kept around 42 °C for more than 30 minutes by using the heating plate (Fig. 8a). Ang II infusion increased systolic blood pressure (SBP) at 7 days and 14 days similarly in AT and AT + heat mice (Fig. 8b). Laboratory data showed that serum ALT (alanine aminotransferase), AST (aspartate aminotransferase) and creatinine were not significantly different among NS, AT and AT + heat groups (Supplemental Fig. 5). These data suggested that repeated hyperthermia stimulation had no effect on SBP and liver and kidney function. Sirius red staining showed that Ang II significantly induced collagen deposition and hyperthermia treatment prevented the fibrotic changes (Fig. 8c). Moreover hyperthermia treatment attenuated Ang II-induced mRNA expression and protein expression of α-SMA (Fig. 8d,e). These data demonstrated that hyperthermia treatment may potentially prevent differentiation of cardiac fibroblasts and cardiac fibrosis in mice heart failure model.

## Discussion

TGF-β is a pleiotropic cytokines that have wide-ranging ability to regulate cell functions such as inflammation, ECM deposition, cell proliferation, differentiation and growth. In addition, TGF-β plays an important role in the pathogenesis of fibrotic conditions *in vivo*^39^. Nevertheless, although it is established that IL-6 production can be induced by β-AR stimulation, Ang II, tumor necrosis factor (TNF)-α, serotonin and adiponectin in cardiac fibroblast from a variety of sources^40^, the present report is the first to show that TGF-β induces IL-6 production in HCFs. We also confirmed that TGF-β promotes α-SMA expression in HCFs, indicating that it induces differentiation of cardiac fibroblasts to myofibroblasts, as previously reported^41^. Importantly, we established here that hyperthermia blocks TGF-β-induced IL-6 production and α-SMA expression in HCFs.

Interestingly, although mild hyperthermia and hyperthermia at 39 °C and 42 °C, respectively, attenuated IL-6 production, only hyperthermia at 42 °C decreased α-SMA and collagen expression. This result indicates that the mechanism (or threshold) regulating IL-6 is different from that regulating α-SMA and collagen. We found that HSP70 was only slightly up-regulated at 39 °C, but was significantly upregulated at 42 °C. This finding is consistent with the report by Kiang *et al*.^42^. Wakisaka *et al*. argued that hyperthermia prevented atrial fibrosis via induction of HSP-72^22^, so it appears that HSPs play a key role in differentiation of HCFs. On the other hand, we showed here for the first time that hyperthermia suppresses the TGF-β1-induced activation of Akt/S6K signaling; *i.e*., Akt/S6K signaling is involved in the anti-fibrotic effect of hyperthermia. The Akt signaling pathway regulates a variety of cellular processes, including survival, proliferation, protein translation and metabolism^43^. Furthermore, the main target of S6K is ribosomal protein S6, a component of the 40 S ribosome subunit, and S6 activation is essential for protein synthesis^38^.

In contrast, the signal transduction pathways that regulate IL-6 gene expression include ERK, p38 MAPK, NF-κB and PI3K/Akt^39^. Our present findings show that hyperthermia attenuated Akt and S6K activation, but not affect Smad2 phosphorylation. This is an interesting result, because numerous studies have shown that the Smad pathway is the dominant signaling pathway regulated by TGF-β^36^. Nevertheless, HT did not prevent Smad2 activation.

To identify the role of Akt signaling, we examined whether Akt inhibitor LY294002 affects the TGF-β1-induced changes of IL-6, α-SMA and collagen expression. LY294002 is a highly selective inhibitor of phosphatidylinositol 3 (PI3) kinase, though it does not inhibit other lipid and protein kinases such as PI4 kinase, PKC, MAP kinase or c-Src at the concentration of 50 μM^44^. We found that LY294002 inhibited all of the above TGF-β1-induced changes in HCFs. It also inhibited Akt/S6K signaling, but did not affect Smad signaling (data not shown). Taken together, our results indicate that the anti-fibrotic effect of hyperthermia is mediated by the Akt/S6K signaling pathway, but not the Smad signaling pathway. These mechanisms are consistent with previous work by Bakin *et al*., who showed that PI3K/Akt signaling is required for TGFβ-mediated epithelial-to-mesenchymal transition and cell migration^38^.

In the past, a cardioprotective effect of hyperthermia has been demonstrated in various animal models. It was proposed that the effect was due to induction of HSP or eNOS and vasodilation by hyperthermia^21,45,46^, and indeed, induction of HSP and eNOS in heart tissue and aortic tissue, respectively, was observed. In contrast, we found that hyperthermia has a direct effect on HCFs activated by TGF-β, blocking the increase of IL-6, α-SMA and collagen expression.

Recent experimental and clinical evidence suggests that chronic inflammation contributes to the pathogenesis of cardiac remodeling and peripheral vascular dysfunction in heart failure. There is considerable evidence that inflammatory cytokines such as IL-6, IL-1 and TNF-α are upregulated in plasma of heart failure patients, as well as in the failing myocardium itself. These mediators are known to be associated with various aspects of cardiac remodeling, such as hypertrophy, fibrosis and apoptosis^47^. Trans-differentiation of cardiac fibroblasts to myofibroblasts is involved in accumulation of excessive ECM^48^. Although cardiac myofibroblasts have an important role in wound healing after heart injury, excessive activation of myofibroblasts contributes to fibrotic change. Therefore, overall, our present and the reported findings suggest that inhibition of IL-6, α-SMA and collagen expression by hyperthermia might be widely applicable for non-pharmacological treatment of cardiac fibrosis and heart failure.

## Electronic supplementary material

Supplementary information
