# Supplementary material for: Acute Hyperthermia Inhibits TGF-β1-induced Cardiac Fibroblast Activation via Suppression of Akt Signaling
Source: Sci Rep. 2018 Apr 19;8:6277. doi: 10.1038/s41598-018-24749-6 (PMC5908876; doi:10.1038/s41598-018-24749-6)
Supplement: Supplementary file 1 — Supplementary information [file 41598_2018_24749_MOESM1_ESM.docx]

**Supplementary information for**

**Acute Hyperthermia Inhibits TGF-β1-induced Cardiac Fibroblast Activation via Suppression of Akt Signaling**

**Masatoshi Narikawa, Masanari Umemura, Ryo Tanaka , Takayuki Fujita, Utako Yokoyama, Tomoaki Ishigami, Kazuo Kimura, Kouichi Tamura and Yoshihiro Ishikawa**

**This PDF file includes:**

Figs. S1, S2, S3, S4, S5, S6, S7, S8, S9 and S10.


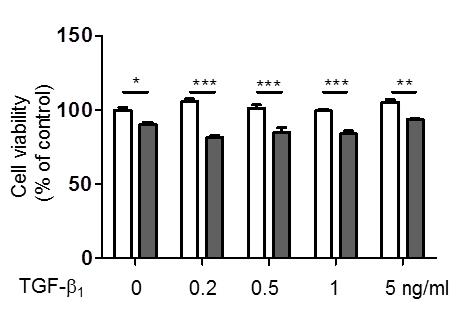


**Supplemental Figure 1.** **Viability of HCFs stimulated by TGF-β1 with (solid bars) or without (open bars) hyperthermia (42 °C)**

(n=5, **p*<0.05, ***p*<0.01, ****p*<0.001).


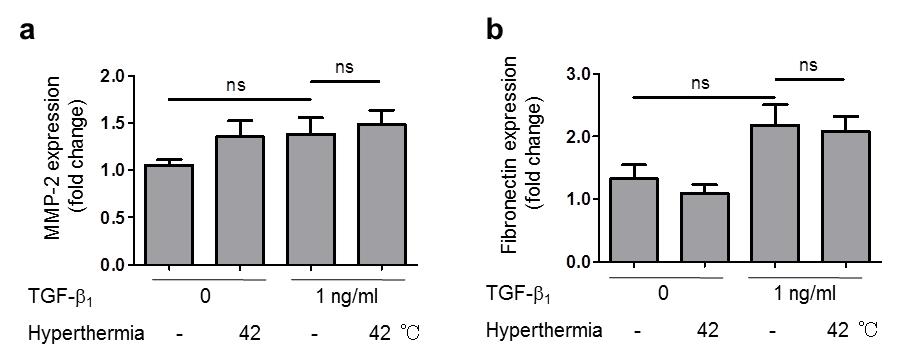


**Supplemental Figure 2**. **Hyperthermia did not affect MMP-2 and fibronectin expression in HCFs.**

(a) MMP-2 mRNA expression in HCFs with or without TGF-b1 for 24 h (n=5, ns: no significant difference). (b) Fibronectin mRNA expression in HCFs with or without TGF-β1 for 24 h (n=4, ns: no significant difference).


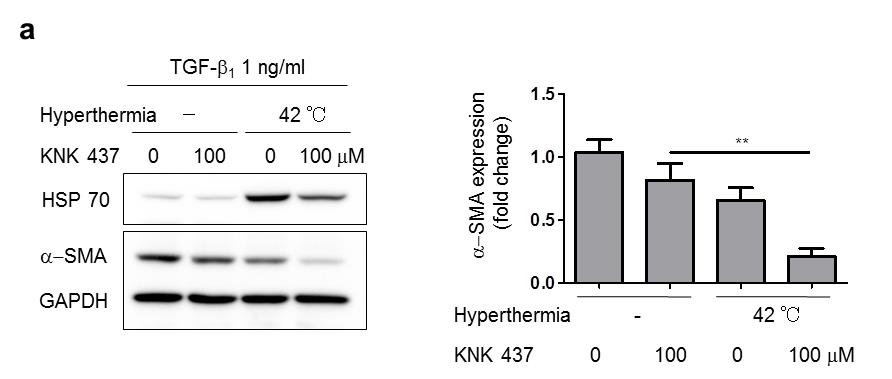


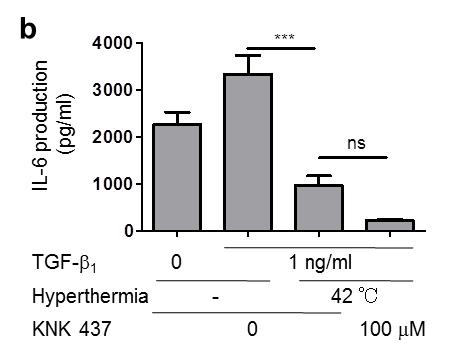


**Supplemental Figure 3**. **HSP70 inhibitor (KNK437) did not influence the effects of hyperthermia on α-SMA and IL-6 expression in HCFs.**

(a) Representative western blot (left) and quantitative analysis (right) of α-SMA expression in HCFs with or without KNK437 for 24 h (n=4, **p<0.01). Original uncropped western blot is shown in Supplemental Figure 10. (b) IL-6 production in supernatant from HCFs with or without KNK437 for 24 h (n=4-6, ***p<0.001, ns: no significant difference).


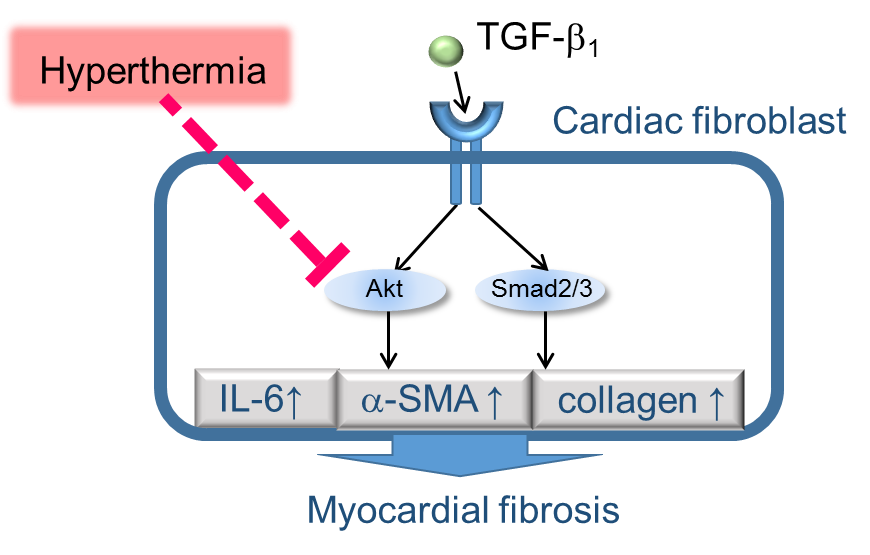


**Supplemental Figure 4**. **Proposed mechanism of anti-fibrotic effect of hyperthermia.** Hyperthermia inhibits IL-6, α-SMA and collagen expression via the Akt/S6K pathway.

**Supplemental Figure 5. Laboratory data on mice at 14 days.**

normal saline (NS), Ang II infusion (AT) or Ang II infusion with hyperthermia treatment (AT + heat), (n=4-5, ns: no significant difference).


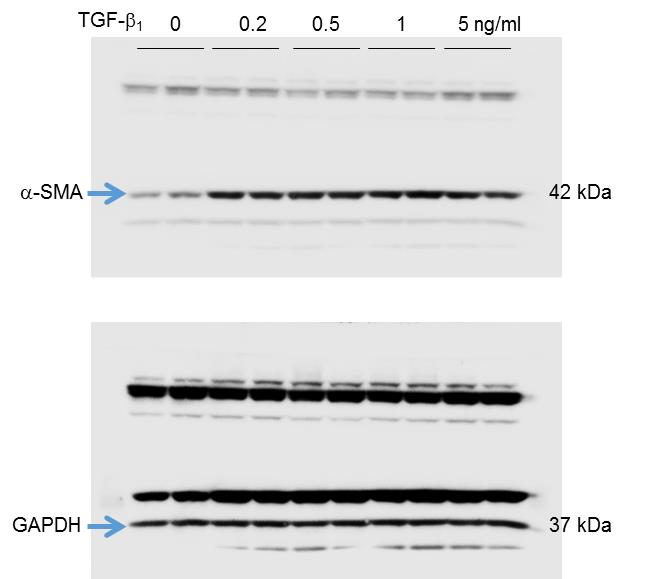


**Supplemental Figure 6. Representative image of the α-SMA protein expression induced by TGF-β1 in HCFs (data shown in Figure 2c).**

**
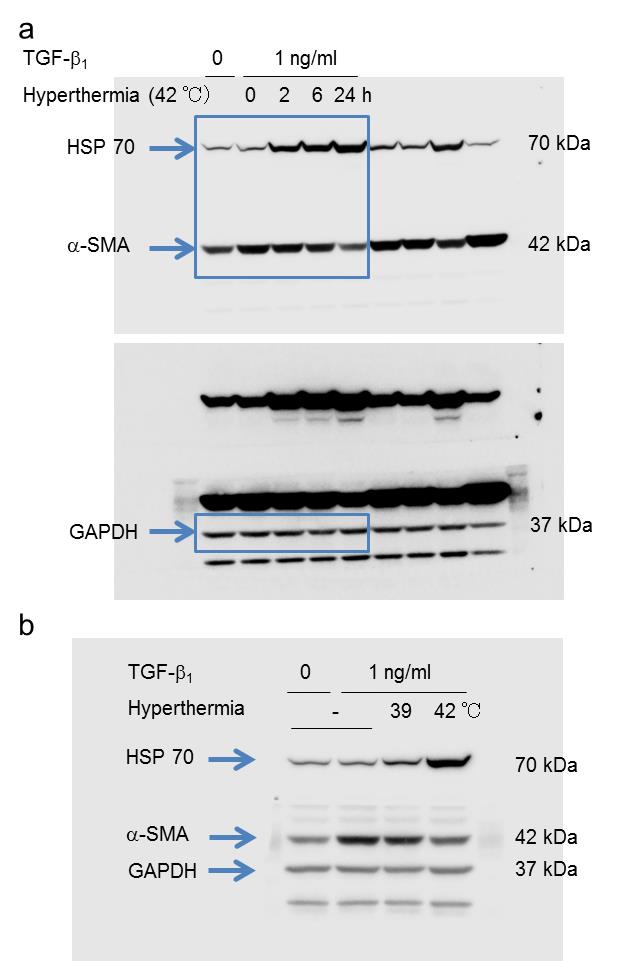
**
**Supplemental Figure 7**. **Representative images of the HSP 70 and α-SMA protein expression induced by TGF-β1 and hyperthermia in HCFs.**(a) is shown in Figure 4b and (b) is shown in Figure 4c.

**
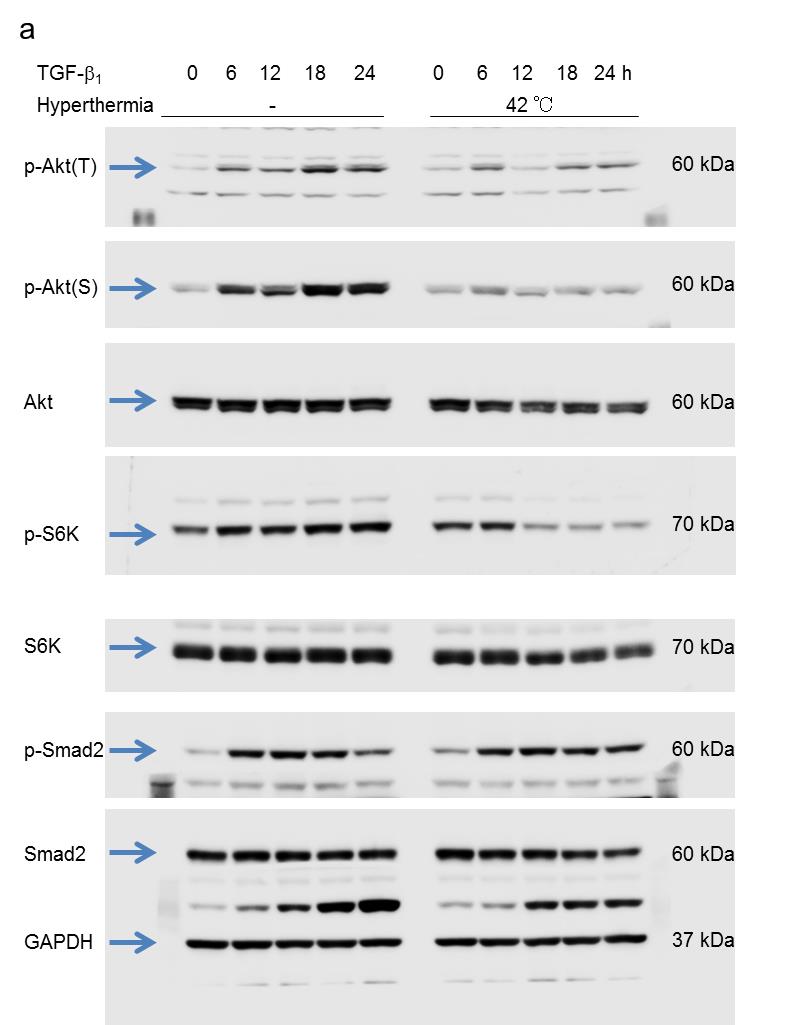
**


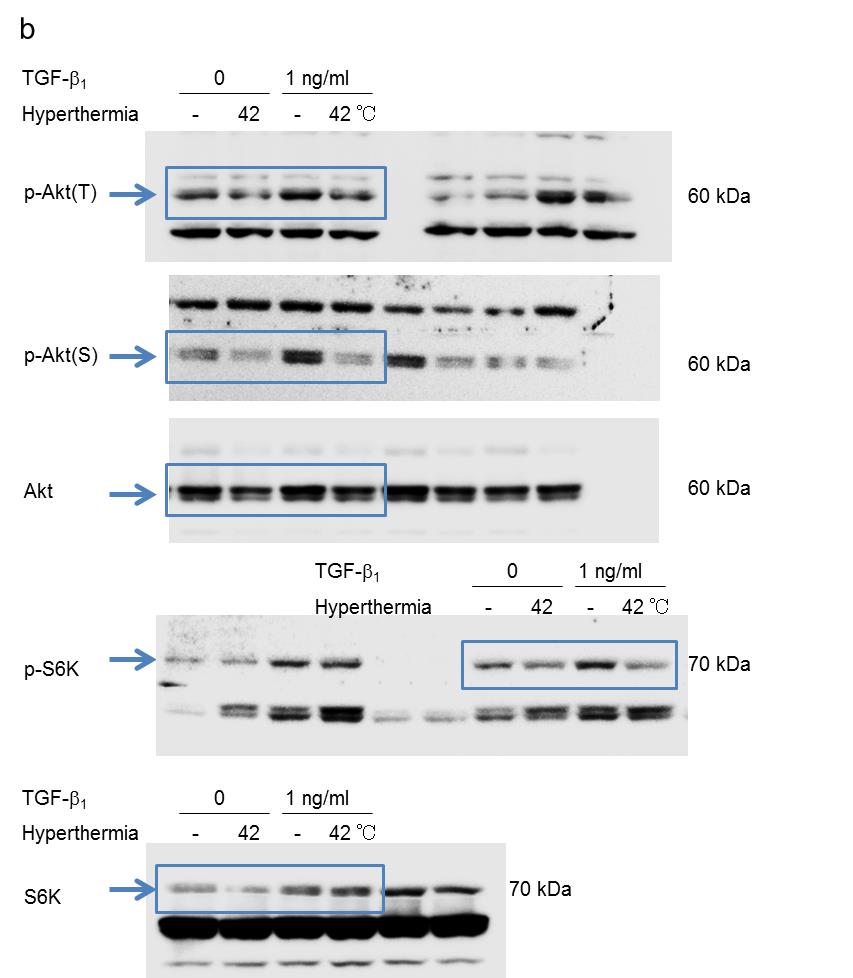


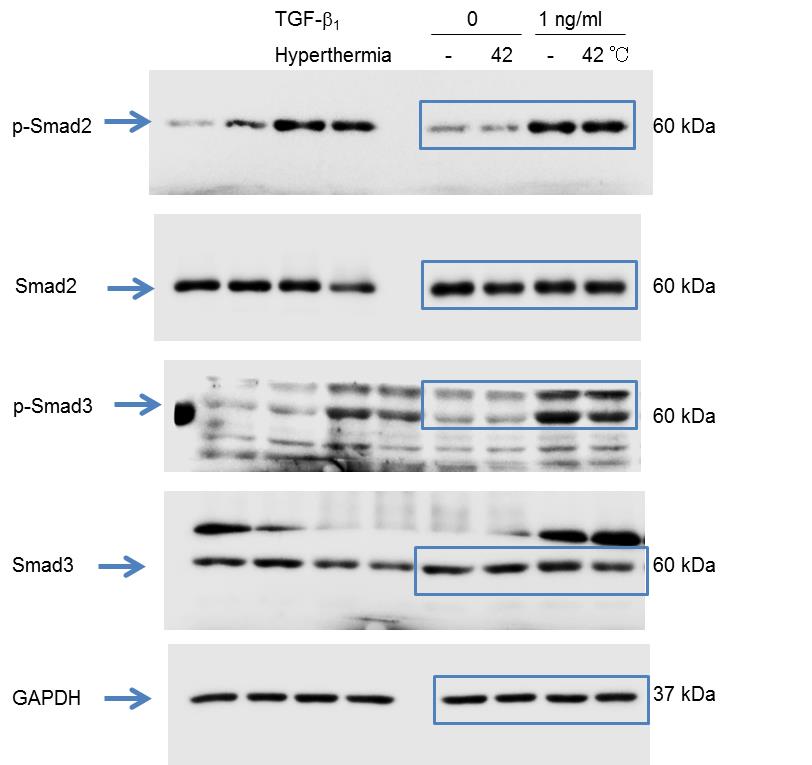


**Supplemental Figure 8. Representative western blots of protein phosphorylation in HCFs stimulated by TGF-β1 with or without hyperthermia.** (a) is shown in Figure 6a and (b) is shown in Figure 6b.


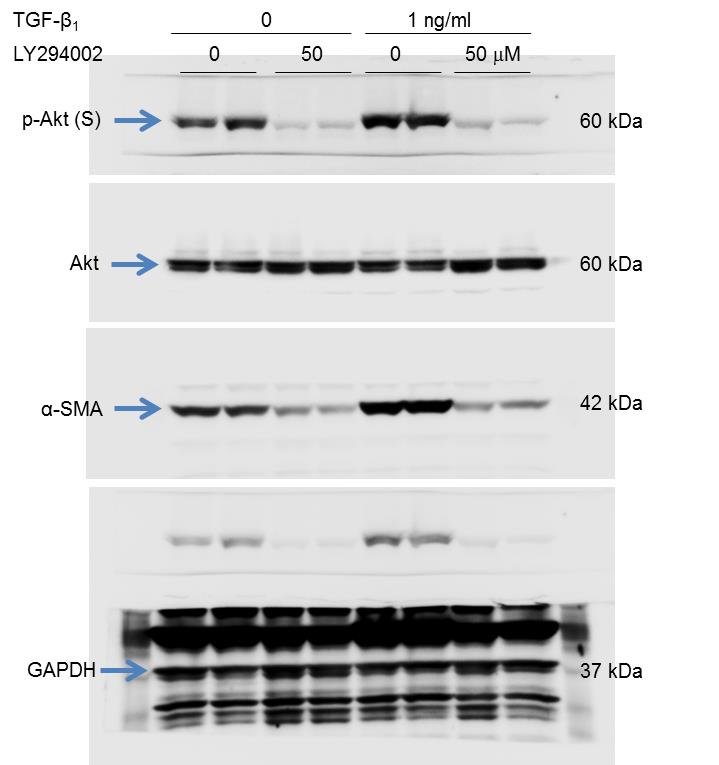


**Supplemental Figure 9. Representative western blots of the p-Akt and α-SMA in HCFs treated by Akt inhibitor(LY 294002) and TGF-β1 (data shown in Figure 7b).**


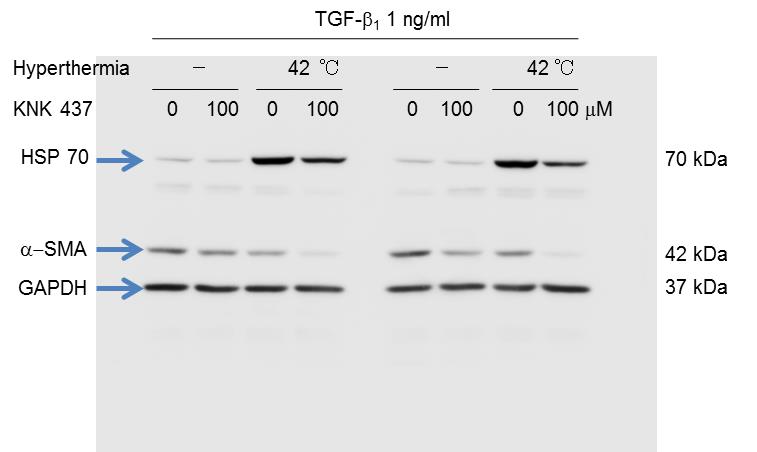


**Supplemental Figure 10. Representative uncropped western blots of HSP 70 and α-SMA in HCFs with or without KNK437 (data is shown in Supplemental Figure 3a).**
